# Field Cancerization in NSCLC: A New Perspective on MicroRNAs in Macrophage Polarization

**DOI:** 10.3390/ijms22020746

**Published:** 2021-01-13

**Authors:** Radu Pirlog, Andrei Cismaru, Andreea Nutu, Ioana Berindan-Neagoe

**Affiliations:** 1 Research Center for Functional Genomics, Biomedicine and Translational Medicine, The “Iuliu Hatieganu” University of Medicine and Pharmacy, 400012 Cluj-Napoca, Romania; pirlog.radu@yahoo.com (R.P.); cismaru_andrei@yahoo.com (A.C.); andreeanutu.an@gmail.com (A.N.); 2 Department of Morphological Sciences, “Iuliu Hatieganu” University of Medicine and Pharmacy, 400012 Cluj-Napoca, Romania; 3 Department of Functional Sciences, Immunology and Allergology, The “Iuliu Hatieganu” University of Medicine and Pharmacy, 400012 Cluj-Napoca, Romania; 4 The Functional Genomics Department, The Oncology Institute “Prof. Dr. Ion Chiricuta”, 400015 Cluj-Napoca, Romania

**Keywords:** lung cancer, field cancerization, tumor microenvironment, macrophage polarization, miRNA

## Abstract

Lung cancer is currently the first cause of cancer-related death. The major lung cancer subtype is non-small cell lung cancers (NSCLC), which accounts for approximatively 85% of cases. The major carcinogenic associated with lung cancer is tobacco smoke, which produces long-lasting and progressive damage to the respiratory tract. The progressive and diffuse alterations that occur in the respiratory tract of patients with cancer and premalignant lesions have been described as field cancerization. At the level of tumor cells, adjacent tumor microenvironment (TME) and cancerized field are taking place dynamic interactions through direct cell-to-cell communication or through extracellular vesicles. These molecular messages exchanged between tumor and nontumor cells are represented by proteins, noncoding RNAs (ncRNAs) and microRNAs (miRNAs). In this paper, we analyze the miRNA roles in the macrophage polarization at the level of TME and cancerized field in NSCLC. Identifying molecular players that can influence the phenotypic states at the level of malignant cells, tumor microenvironment and cancerized field can provide us new insights into tumor regulatory mechanisms that can be further modulated to restore the immunogenic capacity of the TME. This approach could revert alterations in the cancerized field and could enhance currently available therapy approaches.

## 1. Introduction

Worldwide, lung cancer is the leading cause of cancer-related death [1]. In 2018, GLOBOCAN predicted more than two million new cases and 1.76 million deaths, having the incidence almost equal with mortality [1]. Even though, in recent years, there has been significant progress in treatment options for lung cancer patients, the five-year survival has barely increased from 15.6% in 2011 to 19.4% in 2019 [2]. This minor increase in survival is multifactorial, being attributable to decreases in tobacco smoking; increases in early phases detection and advances in treatment strategies (thoracoscopic surgery, radiotherapy, immunotherapy and targeted therapy) [1,3].

Lung cancer is a disease of the elderly, with the median age at diagnosis of 70 years; mortality increases in both genders between the ages of 40 and 85, becoming the primary cause of cancer-related death in men over 40 years and women over 60 years [1,4]. The principal risk factor for lung cancer is smoking, with approximately 90% of cases being related to tobacco use [5]. Tobacco smoke is a complex aerosol composed of more than 5000 compounds, among which at least are 50 are confirmed as carcinogens [6,7,8]. The agents of interests as triggers of lung cancer are the tobacco-specific *N*-nitrosamines, with 4-(methylnitrosamino)-1(3-pyridyl)-1-butanone being the leading promoter of lung cancer [9,10,11]. The mechanisms include the formation of DNA adducts, mutations in tumor oncogenes, free radical damage and cytochrome P450 metabolic activation [11,12,13].

Lung cancer is classified according to the 2015 World Health Organization Classification of Tumors of the Lung, Pleura, Thymus and Heart depending on the tissue of origin in epithelial, mesenchymal lymphohistiocytic, tumors of ectopic origin and metastatic tumors [14]. The two main types of lung cancer refer to epithelial tumors and include non-small cell lung cancer (NSCLC) and small cell lung cancer (SCLC). NSCLC accounts for approximately 85% of the patients and is histologically classified into three main types: adenocarcinoma, squamous cell carcinoma and large cell carcinoma [15,16].

Adenocarcinoma is the most common histologic NSCLC subtype, responsible for approximately 40% of lung tumors. It arises from de novo mutations occurring in the alveolar cells in the peripheric airways [17]. Squamous cell carcinoma represents between 25–30% of the cases, is more centrally located and develops following a multistep process as a progressive reaction to local stressors, leading to hyperplasia, metaplasia, dysplasia and carcinoma [3,18]. Large cell cancers represent 5% to 10% of NSCLC cases and are represented by poorly differentiated tumors composed of large cells that cannot be assigned based on molecular markers to common NSCLC histologic categories [14].

Lung cancer tumorigenesis is a complex, dynamic and, usually, a multistep process based on a combination of genetic predisposition and prolonged exposure to toxins, stressors and environmental pollutants, which, over time, are driving cancer development [18,19,20].

## 2. Lung Cancer Field of Cancerization

Chronic exposure to carcinogens is inducing multistep alterations of the normal respiratory tract, causing various degrees of inflammation and inflammatory-related changes. Over time, acquiring additional mutations in tumor oncogenes and tumor suppressor genes drives cancer progression [21,22,23]. These progressive multistep preneoplastic alterations represent the field cancerization of the respiratory tract [24]. The term was first coined by Slaughter et al. to describe the paraneoplastic adjacent normal-appearing tissue, which gains similar molecular alterations as the primary tumor [25]. Early in tumorigenesis, normal cells accumulate pro-neoplastic mutations that, over time, give rise to a mutant pre-neoplastic clone. The accumulation of additional genetic events by the pre-neoplastic clone promotes the development and progression of cancer [26]. It is essential to recognize that these molecular alterations in the para-neoplastic adjacent tumor tissue are not singular events. However, in a heterogeneous pattern, genetic events are present on variable sizes around the neoplastic process. Cancerized field can display some premalignant changes, such as hyperplasia, metaplasia, different grades of dysplasia or no histologically identifiable changes [27]. For example, specific molecular alterations related to field cancerization were observed using multidiameter single-fiber reflectance spectroscopy at the level of the oral mucosa of lung cancer patients when compared with the oral mucosa of chronic obstructive pulmonary disease control patients [28].

Integrating the field cancerization notion into clinical practice and research raises two immediate consequences. Firstly, after the removal of the primary tumor site, the cancerized field remains in place, with the potential of giving rise to secondary tumors. Secondly, recognizing this prolonged multistep process of gradually acquiring new genetic mutations can provide a window of opportunity for the identification of premalignant phenotypes, allowing us to act before the progression to cancer [29]. Moreover, recognizing the existence of genetic alterations in adjacent para-neoplastic tissue is raising the need for a better selection of normal controls for research, clinical trials and biomarker validation studies, due to high-risk coexisting alterations in tumors and normal adjacent controls, which could hide essential molecular events that could be exploited for critical biomarker or treatment targets [30,31,32].

The lung cancer field of cancerization has been intensively studied, starting from 1996 when Nelson et al. described the first mutations in the *KRAS* genes in nonmalignant adjacent tissue to lung cancer to further studies by Belinsky and colleagues. They reported methylation dysregulation of the *p16* promoter in tumor tissue and normal bronchial epithelium [33,34]. Early studies on NSCLC premalignant lesions identified common aneuploid changes throughout the respiratory tree, suggesting the wide spread of the affected field in the malignant process [35]. A loss of heterozygosity in DNA is observed by genetic analysis in cells obtained from bronchial brushings from both lungs in cancer patients, and the same mutations in the *p53* gene are noticed in the normal bronchial epithelium of cancer-free patients [36,37,38]. Additionally, epigenetic changes were identified in previously thought histological normal adjacent tumor tissue, with common alterations regarding microsatellite instability and CpG island methylation shared across the tumor and adjacent tissues [39]. Studies involving a widespread sampling of the respiratory tract of invasive cancer, carcinoma in situ and high-grade dysplasia patients detected chromosomal alterations inducing allelic imbalances, including oncogenic gains and losses, in tumor suppressor genes supporting the wide spread of field cancerization changes [40]. Mapping of the genome-wide chromosomal alterations in the NSCLC field of cancerization identified allelic imbalances across the respiratory tract pointing to a genomic spatial gradient in the accumulation of mutations, with the main findings including chromosomal loses of regions harboring tumor suppressor genes such as 3p25 (*VHL*), 8p22 (*MTUS1*), 9q (*TSC1*), 19p (*STK11, KEAP1, SMARCA4*) and 13q14 (*RB1*) [41]. The progressive accumulation of genetic events that drives cancer development and progression requires an investigation for biomarkers that can help us distinguish between indolent and potentially malignant pre-neoplastic states [42].

**Figure 1:**
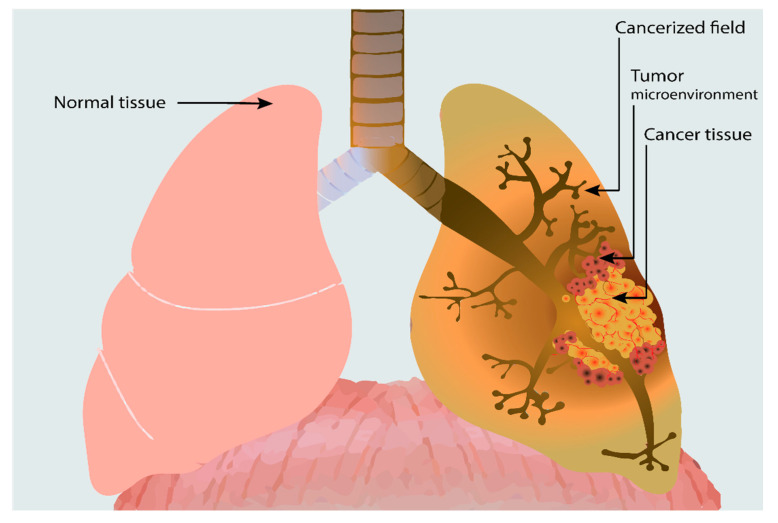
Lung cancer and its associated tumor microenvironment and extensive field cancerizations. We can notice the local spread of the tumor microenvironment (TME) surrounding the tumor, consisting of various cellular populations (lymphocytes, neutrophils, macrophages, dendritic cells, fibroblasts, etc.). Adjacent to the TME, the cancerized field is associated with lung cancer, which diffusely extends upwards in the respiratory tract. The cancerized field can present various histologic changes, from hyperplasia to metaplasia and high-grade dysplasia, or might appear histologically normal, bearing only some focal genetic alterations.

The notion of field cancerization, which represents the paraneoplastic adjacent normal-appearing tissue that gains similar molecular alterations, as in the primary tumor [25], needs to be distinguished from the tumor microenvironment (TME) that consists of a heterogeneous cellular environment surrounding and infiltrating the tumor that is composed roughly of cellular immune populations, supporting stroma and intercellular mediators (Figure 1) [43].

Field cancerization and TME are sometimes used interchangeably, leading to confusion. To better characterize these two notions, we summarized a comparative analysis to highlight the individual characteristics of the two entities in Table 1.

## 3. Lung Cancer and Tumor Microenvironment

In the early phases of cancer progression, the malignant niche generates its tumor sanctuary by attracting cellular populations from the bloodstream to the TME [43,50]. The TME is a complex structure consisting of immune and structural components. The immune component consists of heterogeneous populations of inflammatory cells, such as T lymphocytes, B lymphocytes, natural killer cells, natural killer T cells, cancer-associated fibroblasts (CAF) and tumor-associated macrophages (TAM) [43]. The structural component of the TME, the stroma, is a dual entity composed of a cellular compartment (CAFs, TAMs, endothelial cells, pericytes, adipocytes and bone marrow mesenchymal stromal cells) [49,51] and a noncellular compartment, which includes various molecules circulated between TME and tumor cells [52].

TME is a hypoxic medium due to a high cellular density and scarce oxygen supply [53]. Cancer cells and TME cellular populations undergo phenotypic changes that translate into the generation of high amounts of reactive oxygen species (ROS) and hypoxia-inducible factor 1α (HIF-1α), which modulate the molecular and phenotypic dynamic events taking place in the cancer hypoxic environment [54,55]. HIF-1α is an essential regulator of oxygen homeostasis involved in pro-neoplastic events taking place in the hypoxic TME. It is directly involved in the modulation of essential processes such as cancer progression, epithelial-to-mesenchymal transition (EMT), angiogenesis and metastasis [54,56,57]. The hypoxic environment in the TME contributes to early tumorigenesis and supports the neoplastic tumor signaling pathways, thus enabling cancer niche proliferation, survival and metastasis [58]. Chronic exposure to a hypoxic environment, ROS and high amounts of HIF-1α leads to a progressive genetic and phenotypic transformation of normal stromal cellular populations, contributing this way to the field cancerization induced by the neoplastic process [55]. CAFs are a major cellular component of the TME, being recruited from different stromal cells [59]. CAF recruitment is a process dependent on the existence of ROS at the TME level, which becomes self-sustaining, as CAFs promote the hypoxic environment by becoming major ROS generators [60]. The formation of this pro-oxidative state is essential in the early phases of TME development, as it facilitates the recruitment of other cellular populations into the tumor sanctuary and promotes acquiring the hallmarks of cancer by the neoplastic niche [59].

TME is essential for the acquisition of the hallmarks of cancer by the primary tumor. In the last decades, advances into the understandings of the complex cellular interactions at the TME level resulted in the decoding of the programmed cell death protein 1 (PD1)/ programmed cell death protein-ligand 1 (PD-L1) pathways, leading to the development of immunotherapy, restoring the TME immunogenic potential [49,61].

Between tumor cells and TME is a continuous multidirectional communication via the exchange of biomolecules (proteins, microRNA (miRNA), long noncoding RNA (lncRNA) and mRNA) that are transferred through extracellular vesicles (EVs) [62,63]. EVs represent an effective communication method for regulatory and metabolic cellular processes, being considered major players in tumor development [63,64]. Biomolecules regulate the interplay between immune cells and other TME constituents, being exchanged directly or through EV, among which, miRNAs represent the main component. MiRNAs have essential roles in modulating the expression levels of important genes involved in critical neoplastic cellular processes, including proliferation, apoptosis, autophagy, cellular senescence and non-self-recognition [63,65,66].

TAMs represents a prominent immune population in TME, their density being associated with a poor prognosis [67]. During cancer progression, monocytes are recruited from the bloodstream, and under the influence of local environmental conditions, they differentiate into TAMs that enhance tumor progression by promoting angiogenesis, invasion and metastasis [67,68]. Under the influence of paracrine molecular signals from tumor cells and cellular components of the TME, differentiated monocytes adopt two phenotypes: M1 (classically activated macrophages) and M2 (alternatively activated macrophages), a process known as macrophage polarization [69]. M1 macrophages are driven by the Th1 cytokine interferon (IFN-γ) and express proinflammatory factors such as interleukin-6 (IL-6), IL-12 and IL-23, being potent antitumoral cells. M2 macrophages upregulate the expression of macrophage mannose receptors (CD206), scavenger receptors and CD163, as well as M2-type immunosuppressive cytokines (IL-10) [70,71]. A retrospective study on TAMs in lung cancer showed that 70% of macrophages have an M2 phenotype and that the density of the remaining 30% of M1 macrophages represents an independent predictor of survival [72]. The prognostic difference can be attributed to M2 macrophages that lose the cytotoxic ability and promote pro-neoplastic processes, including cancer-related inflammation, immunosuppression, angiogenesis, tissue remodeling and metastasis [73].

Macrophage polarization is a dynamic bidirectional process that takes place under the influence of various local factors [74]. It can be artificially induced towards an M2 phenotype by enhancing the pro-oxidative metabolism and promoting the M1 phenotype by inhibiting oxidation [75]. The presence of a hypoxic environment in the TME and metabolic differences of the M1/M2 macrophage phenotypes represent a complex, tightly regulated phenomenon influenced by various features of tumor and TME cells involved in regulating polarization [74,76]. M1 macrophages have an oxygen-dependent metabolism based on glycolysis, while M2 macrophages are present in hypoxic areas of the tumor being involved in angiogenesis [77].

## 4. miRNAs Roles in Lung Cancer TAM Polarization

**Figure 2:**
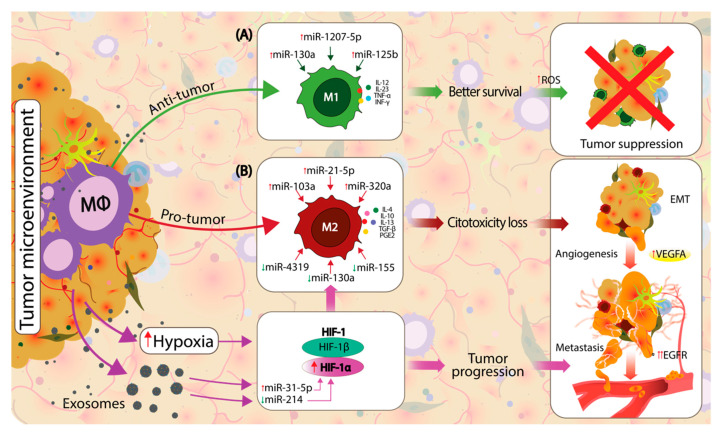
Macrophage polarization in the TME is influenced by local factors towards an M1 proinflammatory, antitumor or towards an M2 protumor, anti-inflammatory state. Cancer and TME cells directly influence the macrophage phenotypic dynamics by modifying the composition of secreted exosomes, which circulate the noncoding RNAs (ncRNAs), microRNAs (miRNAs) and other molecules between cells. (**A**) The upregulation of miR-130a, miR-1207-5p and miR-125b direct the macrophage polarization towards an M1 phenotype in non-small cell lung cancer (NSCLC), which will express M1 proinflammatory markers such as interleukin (IL)-12, IL-23, TNF-α and interferon gamma (IFN-γ), which further increase the reactive oxygen species (ROS), leading to efficient tumor suppression. The local TME is a hypoxic environment that stimulates the upregulation of *hypoxia-inducible factor 1 (HIF-1)* genes. Hypoxia-inducible factor 1-alpha (HIF-1α) is upregulated by local hypoxia and by an upregulation of miR-31-5p and downregulation of miR-214. HIF-1α is a known modulator of M2 macrophage polarization. (**B**) Polarization towards the M2 phenotype is acquired by the direct influence of HIF-1α and various dysregulated miRNAs from the local TME, including the upregulation of miR-103a, miR-21-5p and miR-320a and downregulation of miR-4319, miR-130a and miR-155. M2-polarized macrophages express specific markers, such as IL-4, IL-10, IL-13, TGF-β and PGE2, which further support the pro-neoplastic processes. HIF-1α and M2 macrophages support the tumor progression by enhancing the epithelial-to-mesenchymal transition (EMT), angiogenesis and metastasis.

An emerging class of molecules involved in regulating the M1/M2 balance is represented by miRNAs [78,79,80]. MiRNAs are 19–25-nt short noncoding RNA sequences that modulate gene expression programs by influencing the translation and stability of target mRNA [81]. Studies focus either on individual miRNAs characterized concerning different oncogenic pathways or as a combination of miRNAs shown to modulate macrophage phenotype transitions in lung cancer [82,83]. In NSCLC, the M1/M2 balance is modulated by differently expressed miRNAs secreted by TME and tumor cells (Figure 2) [84,85]. The current view on the M1/M2 balance suggests that the present macrophages are in a continuum phenotypic dynamic, making it difficult to appreciate their prognostic role in NSCLC [86,87]. A better understanding of how different miRNAs could influence the M1/M2 balance could influence the development of novel targeted therapy approaches that aim to re-educate the macrophages towards the M1 phenotype [74].

In lung cancer, miRNAs have critical regulatory roles in essential cancer metabolic pathways, including proliferation, angiogenesis, invasion and metastasis [88,89,90,91]. MiRNA expressions were found to be dysregulated in the bronchial epithelium of cigarette smokers and lung cancer patients compared with normal nonsmoker controls, supporting the existence of a molecular dysregulation affecting the whole respiratory tract [92,93,94]. Pavel et al. proposed a panel of four miRNA (miR-146a-5p, miR-324-5p, miR-223-3p and miR-223-5p), which are specifically downregulated in the bronchial epithelium of NSCLC, supporting the existence of a field of injury in lung cancer that can be characterized by molecular analysis [94,95]. Molecular differences in smoker and nonsmoker bronchial epitheliums were further analyzed by high-throughput arrays that identified differentially expressed genes related to antioxidant roles and alterations in miRNAs expressions [94,96,97]. Furthermore, a recently combination of genes were proposed as signature models to distinguish neoplastic-transformed malignant epithelium to normal bronchial epithelium [98].

Hypoxia increases the number of vesicles secreted by resident cells of the TME and alters the compounds of these EVs [62,64]. Mesenchymal stem cells exposed to hypoxic environmental conditions increase the EV secretion and enrich the EV composition with hypoxia-induced miRNAs, including miR-21, miR-210, miR-23a, miR-370, miR-373 and miR-103a, which regulate the tumor immune response and are directly involved in cancer development [62,99]. As a response to hypoxia, HIFs genes are activated and modulated by various miRNAs secreted by the tumor and TME cells. HIF-1α upregulation is induced by a downregulation of miR-214 and upregulation of miR-31-5p. This genetic upregulation of HIF-1α increases the vascular endothelial growth factor (VEGF) and promotes a metabolic switch in cancer and TME cells towards glycolysis (Warburg effect), which, in turn, promotes tumor angiogenesis and proliferation [100,101]. The downregulation of HIF-1α by the upregulation of miR-130a, miR-155, miR-199a and miR-200c have opposite effects by proliferation, invasion and metastasis [102,103,104,105]. Hypoxic conditions were found to trigger the macrophage M2 phenotype transition through the direct modulation of miRNAs such as miR-301a-3p, miR-940, miR-21-3p, miR-125b-5p and miR-181d-5p [106,107].

The intercellular transfer of EVs from hypoxic lung cancer was shown to modulate macrophage polarization through miR-103a towards the M2 phenotype. A research study focused on hypoxic lung cancer cell lines NCI-H2087, NCI-H1792 and NCI-H1437 by Hsu et al. showed that these cell lines secreted EVs with a high content of miR-103a, which influenced the macrophage polarization [82]. The hypoxic conditions dysregulated the EV constituents by upregulating miR-130a which inhibits *Phosphatase And Tensin Homolog (PTEN)* expression, a known tumor suppressor [82,85]. *PTEN* modulates macrophage polarization through the Phosphoinositide-3 kinase/protein kinase-B (PI3K/AKT) signaling pathway [108]. The downregulation of *PTEN* via miR-130a increases AKT and Signal transducer and activator of transcription 3 (STAT3), leading to the accumulation of M2 macrophages (CD163+CD206^high^HLA-DR^low^ cells) and increases the expression of prooncogenic factors [82,108].

MiR-21 is known as an oncogenic miRNA in various cancers, including NSCLC, where its upregulation is associated with a worse prognosis. A deregulated miR-21 expression influences critical oncogenic pathways, including proliferation, angiogenesis and metastasis [109]. Mir-21 was identified as the most abundant miRNAs in TAMs, with its downregulation leading to a proinflammatory M1-type phenotype via the increased expression of *mitogen-activated protein kinase-3* gene, the induction of p38-CHOP and cJun-NH2-terminal kinase signaling [110]. Yang et al. systematically analyzed the literature regarding miR-21 and NSCLC, identifying that a high expression of miR-21 negatively impacts the overall survival (HR = 2.32 (1.17–4.62), *p* < 0.05) [111]. Furthermore, a hypoxic environment was shown to stimulate miR-21 secretion by human bone marrow-derived mesenchymal stem cells towards macrophages, increasing the M1/M2 balance towards an M2 phenotype. This phenotypic switch promotes cancer survival and proliferation in cell lines through the downregulation of PTEN, programmed death cell ligand 4 (PDCD4) and Reversion-inducing cysteine-rich protein with Kazal motifs (RECK) [85]

MiR-155 is a known oncogenic miRNA studied in multiple cancers. Its expression is dysregulated in multiple solid and hematological malignancies [54,112,113,114]. A meta-analysis by Shao et al., investigating the value of miR-155 as a diagnostic and prognostic biomarker in lung cancer, identified miR-155 as a promising circulating biomarker for lung cancer, being upregulated in the serum of lung cancer patients. However, corroborating results from recently published studies failed to predict a significant prognostic role for miR-155 in lung cancer; analyses showed a 1.26-fold higher risk for a poor overall survival (OS) [113]. MiR-155 overexpression is associated with chemotherapy resistance by forming a feedback loop with *TP53* that can be successfully targeted to overcome treatment resistance [115]. Huang et al. analyzed the effects of Cypermethrin (CYM), a type II pyrethroid, on macrophage polarization and the implications of a M2 phenotypic shift in lung cancer progression and metastasis. They showed that CYM induces the downregulation of miR-155, which enhances *B-cell lymphoma 6 (Bcl6)* expression, decreases the expression of mitogen-activated protein kinase 4 (MKK4) and inhibits c-Jun N-terminal kinases (JNK) activation. These genetic alterations result in an inhibition of M1 polarization, activation of metastasis-related genes and promotion of the alternative M2 activation and tumor metastasis [116].

Initial reports on the miR-1207 role in cancer biology described its role as a tumor suppressor miRNA by targeting the telomerase reverse transcriptase and, thus, suppressing the proliferation and invasion in gastric cancer [117]. An in vitro study on EMT using nasopharyngeal cancer cells identified miR-1207 among the miRNAs involved in the regulation axis of EMT. MiR-1207 upregulation is able to suppress EMT and the metastasis phenotype of cancer cells [118]. Dang et al. showed that miR-1207 has an extensive role in EMT by the direct regulation of key proteins such as Snail, Smad2, Smad3, Smad7, Vimentin and Zinc finger E-box-binding homeobox 1 (ZEB1), making it an important regulator of tumor invasion and metastasis. They further explored the miR-1207 role in NSCLC and identified the *Colony-stimulating factor 1 (CSF1)* as its target gene. CSF1 is a hematopoietic growth factor involved in the differentiation, proliferation and survival of macrophages, being a significant regulator of TAMs. The upregulation of miR-1207-5p in macrophage d-THP1 cells switched the phenotype balance towards M1 by increasing IL-12 and IL-23 and significantly reduced the expression of the M2 features IL10 and VEGF. In vitro studies on NSCLC cell lines showed that the upregulation of miR-1207-5p could inhibit CSF1 mRNA/protein expression and reduce proliferation, migration and invasion. MiR-1207-5p further downregulated the downstream targets in the CSF1 signaling pathway, inducing the downregulation of STAT3 and AKT. Based on in vitro study results, Dang et al. developed a xenograft nude mice mouse model that showed that miR-1207-5p reduces lung cancer metastasis [119].

MiR-320a is deregulated in several cancers, including gastric, colon and lung cancer [120,121]. In NSCLC, it was described as a tumor suppressor acting on insulin growth factor (IGF)-1R53, being involved in tumor growth and metastasis through STAT3 and Neuropilin 1 downregulation [122,123,124]. Fortunato et al. used a combined in vivo and in vitro approach using tissue, blood and cell lines to analyze the miRNA role in the development of a protumorigenic microenvironment and their immunity modulation in cancer progression. In vitro studies on M1/M2 macrophage polarization, using miRNAs profiles, identified the upregulation of miR-15b, miR-197 and miR-320a-promoted M2 polarization. MiR-320a had a higher expression level during polymorphonuclear neutrophils activation, supporting its role in macrophage polarization. The upregulation of miR-320a inhibited STAT4 expression in macrophages, promoting polarization towards a M2 phenotype [83]. The STAT protein family are essential players in macrophage polarization, with STAT1, STAT3 and STAT6 having clearly defined roles in the process by promoting inflammatory responses and mediating the transcription of key interleukins [123,125]. A research paper by Fortunuato et al. showed that activated neutrophils in heavy smokers increased their EV-mediated miR-320a secretion towards macrophages, which promoted polarization towards a M2 phenotype through the downregulation of STAT4. Furthermore, these polarized macrophages had oncogenic effects on the A549 lung cancer cell lines, increasing the invasiveness and migration through VEGF upregulation [83].

MiR-130a roles were described in multiple cancer pathogenesis, including hepatocellular, cervical, ovarian and lung cancer. It is an essential player in acquiring drug resistance as a modulator of the PI3K/Akt/PTEN/ mechanistic target of rapamycin (mTOR), Wnt/β-catenin and NF-kB/PTEN drug resistance signaling pathways [126]. A study involving 75 NSCLC and the corresponding controls identified lower levels of miR-130a in tumor tissue than in the adjacent controls. Moreover, the downregulation of miR-130a correlated with a higher tumor stage and tumor node metastasis, supporting its role as a tumor suppressor. The same team analyzed the role of miR-130a on macrophage polarization using the THP-1 cell lines. They showed that miR-130a expression levels are dependent on the M1/M2 phenotype. miRNA upregulation favors a polarization towards the M1 proinflammatory phenotype and downregulation towards M2. This phenotype switch is induced by the direct miR-130a inhibition of Proliferator-activated receptor γ (PPARγ) [84]. PPARγ is a transcription factor involved in lipid metabolism and energetic homeostasis. PPARγ is known for its inhibitory role on proinflammatory genes, and PPARγ activation is considered an essential step towards M2 macrophage polarization [127].

MiR-125b is an essential modulator of cancer tumorigenesis, being involved in macrophage activation and the modulation of various oncogenic pathways, including NF-κB, p53 and PI3K/Akt/mTOR, having significant roles in modulating the essential cancer pathways, including proliferation, apoptosis, metastasis and drug resistance [128]. The MiR-125b role on macrophage polarization was analyzed in an in vivo study using genetically engineered naive and *KRAS/p53* double-mutant NSCLC mouse models treated with CD44-targeting hyaluronic acid-poly(ethylenimine)based nanoparticles (HA-PEI) encapsulating miR-125b. Parayath et al. showed that the upregulation of miR-125b switches the macrophage balance towards the M1 phenotype. These intraperitoneally administered HA-PEI nanoparticles are a reliable model that could be further investigated as an effective strategy to repolarize the TME towards a proinflammatory phenotype [129].

Moreover, besides the role of miRNA in the modulation of TAM, new evidence supports the existence of complex axes involving interactions between long noncoding RNAs (lncRNAs), miRNAs and target genes [130]. LncRNAs are noncoding RNA sequences longer than 200-bp RNAs with important known modulatory roles such as DNA damage, miRNA silence, inflammation and tumorigenesis [131,132]. Li et al. analyzed GNAS-AS1, a lncRNA with an emerging role in the cancer biology of NSCLC and associated with TAM polarization. They analyzed NSCLC tumor tissue and adjacent controls, identifying a negative correlation between GNAS-AS1 expression with overall survival and metastasis-free survival. In vitro studies on the NSCLC and THP-1 cell lines showed that GNAS-AS1 is upregulated in the TAM and NSCLC cell lines. Additionally, they showed that GNAS-AS1 upregulation directly inhibits miR-4319, which leads to the upregulation of the N-terminal EF-hand calcium-binding protein 3 (NECAB3) protein level. Li’s study described a novel axis involving GNAS-AS1/miR-4319/NECAB3 that negatively influenced the NSCLCL prognosis by influencing the TAM polarization towards an M2 phenotype [130].

## 5. Clinical Signification of Field Cancerization and Macrophage Polarization

Being aware of the extensive changes that occur in the respiratory tract of high-risk heavy smokers and lung cancer patients offers new insights into the multistep alterations leading to field cancerization of the respiratory tract. These progressive multistep changes occurring at the level of whole systems alter the normal structure of the stroma, facilitating the engrafting and proliferating of possible neoplastic niches [133]. In the case of familial adenomatous polyposis, an autosomal-dominant syndrome caused by genetic alterations in the *adenomatous polyposis coli* (*APC*) gene, patients have the genetic alteration present at the level of the entire colic tract. This genetic alteration promotes field cancerization of the colonic tract, leading to an altered protumorigenic colonic soil fertile for the engrafting of cancer-primed cells.

New insights into the role of soil in the engrafting and promoting of oncogenesis were presented by Li et al., who showed that, in the case of familial adenomatous polyposis patients, spatially separated tumors can occur from the same cancer cell, highlighting the important role of primed soil in developing additional tumors [134]. A better understanding of the molecular shifts taking place at the cellular level could identify the specific molecular profile of the cancerized field, allowing to identify individuals at risk of developing cancer. Advances into molecular shifts taking place at the cellular level in patients developing pre-neoplastic lesions can be identified and stratified according to their risk of progressing towards cancer by identifying specific signatures; one approach is by a miRNA profile of the cancerized field. [135].

A set of elegant experiments by Danilov et al. which analyzed the conformational change in the structure of the Angiotensin I-Converting enzyme by using a panel of 16 monoclonal antibodies in lung cancer and normal controls, was able to identify a signature for early stage lung cancer field cancerization. Their experiments identified specific conformational changes taking place in these patients, offering new insights into early molecular changes that “prepare the soil” for the neoplastic niche and, at the same time, offering new opportunities for early cancer screening, risk stratifying and possible treatment targets [136].

## 6. Conclusions

Technological advances allow us to understand molecular alterations that take place at the cellular level and to characterize the spatial distribution of the progressive alterations from the normal to premalignant states and to cancer. Currently, especially in NSCLC, we need to integrate into research and clinical practice the notions of field cancerization and TME with their specific characteristics.

Field cancerization in lung cancer extends in the respiratory tract at all levels due to chronic exposure to toxins that are able to reestablish tumor progression after the initial tumor removal. From a research perspective, integrating the notion of field of cancerization into experiment designs is essential when looking for normal controls, searching for biomarkers or studying the interrelation between tumor cells, TME and adjacent normal tissue.

A better understanding of the molecular aspects of the cancerized field can offer new opportunities to screen for alterations that take place after surgical interventions, to evaluate the histologic and molecular changes and to specifically act upon the cancerized field to diminish the risk for secondary cancers. The TME surrounds and infiltrates the tumor and consists of various heterogenic cellular populations, among which, TAM represents a major subpopulation that undergoes dynamic phenotypic changes based on the molecular composition of the TME. Thus, various miRNAs were described through in vitro and in vivo studies that can polarize TAMs towards proinflammatory or anti-inflammatory states. This dynamic phenotype occurring at the TAM level should be further investigated to develop therapeutic strategies that shift macrophage phenotypes. These cellular therapies could be further joined with the currently available chemotherapy and radiotherapy regimens to enhance the therapeutic effect.

Understanding the interrelation between the cancerized field, TME and phenotype switches that take place at the cellular level can drive novel chemoprevention strategies that will be able to reprogram the TME to enhance the immunogenic properties of the cellular component. The complex interactions involved in macrophage polarization, although intricate, support the theory that the cellular phenotype and interactions present at the level of the TME can be modulated by miRNAs to enhance the immunogenic capacity of the TME. This approach needs further investigation, as it could have important implications for cancer immunotherapy, with miRNAs being used as predictive biomarkers or enhancers of the currently available therapies.

## Figures and Tables

**Table 1:** Lung cancer field cancerization and tumor microenvironment comparative analysis.

	Field Cancerization	Tumor Microenvironment	Ref
**Localization**	Widespread in the respiratory tract.	Peritumoral and intratumoral	[43,44]
**Main characteristics**	Progressive DNA damage. Accumulation of oncogenic mutations. Inactivation of tumor suppressor genes.	Epithelial–mesothelial transition activation, heterogenous cellular populations, complex roles in tumorigenesis and acquiring of the hallmarks of cancer by the tumor.	[25,26,43,45]
**Pathology**	Adjacent premalignant lesions are identifiable on classic histology. Normal-appearing adjacent tissue might harbor genetic mutations that need further molecular analysis.	Identifiable with classic histology and with immunohistochemistry for a specific cellular population.	[46,47]
**Removal**	Not removed with the primary tumor and can give rise to second tumors.	Usually removed with the main tumor.	[48]
**Constituents**	Normal-appearing cells or premalignant cells of the respiratory tract.	Immune populations composed mainly of leucocytes and macrophages and stromal populations, including the extracellular matrix, blood vessels and signaling molecules.	[25,44,45]
**Role(s)**	Develops due to progressive exposure to stressors and toxins and can progress and give rise to tumors.	Supports tumor progression and development and creates a tumor sanctuary that promotes proliferation and dissemination.	[44,45,46,49]

## Data Availability

Not applicable.

## Abbreviations

APC	Adenomatous polyposis coli
Bcl6	B-cell lymphoma 6
CAF	Cancer-associated fibroblasts
CSF	Colony-stimulating factor 1
CYM	Cypermethrin
HR	Hazard Ratio
IL	Interleukin
IGF	Insulin Growth Factor
JNK	c-Jun N-terminal kinases
LncRNAs	Long noncoding RNAs
miRNAs	microRNAs
MKK4	Mitogen-activated protein kinase 4
mTOR	Mechanistic target of rapamycin
ncRNAs	Non-coding RNAs
NSCLC	Non-small cell lung cancer
NECAB3	N-terminal EF-hand calcium-binding protein 3
PD1	Programmed cell death protein 1
PD-L1	Programmed cell death protein-ligand 1
PDCD4	Programmed death cell ligand 4
PPARγ	Proliferator-activated receptor γ
PTEN	Phosphatase And Tensin Homolog
RECK	Reversion-inducing cysteine-rich protein with Kazal motifs
ROS	Reactive oxygen species
SCLC	Small cell lung cancer
STAT	Signal transducer and activator of transcription
TAM	Tumor-associated macrophages
TME	Tumor microenvironment
VEGF	Vascular endothelial growth factor
ZEB1	Zinc finger E-box-binding homeobox 1
